# Metabolic Biomarkers for Prognostic Prediction of Pre-diabetes: results from a longitudinal cohort study

**DOI:** 10.1038/s41598-017-06309-6

**Published:** 2017-07-26

**Authors:** Hailuan Zeng, Renchao Tong, Wenxin Tong, Qiaoling Yang, Miaoyan Qiu, Aizhen Xiong, Siming Sun, Lili Ding, Hongli Zhang, Li Yang, Jingyan Tian

**Affiliations:** 1 Department of Endocrinology and Metabolism, Shanghai Institute of Endocrine and Metabolic Diseases, Ruijin Hospital, Shanghai Jiao Tong University School of Medicine, Shanghai, China; 2 The MOE Key Laboratory for Standardization of Chinese Medicines, Institute of Chinese Materia Medica, Shanghai University of Traditional Chinese Medicine, Shanghai, China; 3 Department of Diabetes Complications and Metabolism, Beckman Research Institute, City of Hope, 1500 East Duarte Road, Duarte, CA USA; 4 Center for Chinese Medical Therapy and Systems Biology, Shanghai University of Traditional Chinese Medicine, Shanghai, China

## Abstract

To investigate the metabolic biomarkers of predicting the transition from pre-diabetes (pre-DM) to normal glucose regulation (NGR) and diabetes (DM) in a longitudinal cohort study. 108 participants with pre-DM were followed up for ten years and divided into 3 groups according to different glycemic outcomes. 20 participants progressed to DM, 20 regressed to NGR, and 68 remained at pre-DM. Alterations in plasma metabolites in these groups were evaluated by untargeted ultra-performance liquid chromatography-quadrupole time-of-flight mass spectrometry (UPLC-QTOF-MS). Twenty three metabolites related to glycerophospholipid metabolism, oxidation and antioxidation were associated with the process from pre-DM to NGR, while twenty two metabolites related to amino acid metabolism, glycerophospholipid metabolism and mitochondrial β-oxidation played important roles in the progression to DM. Results from stepwise logistic regression analysis showed that five biomarkers (20-Hydroxy-leukotriene E4, Lysopc(20:4), 5-methoxytryptamine, Endomorphin-1, Lysopc(20:3)) were good prediction for the restoration to NGR, and five biomarkers (Iso-valeraldehyde, linoleic acid, Lysopc(18:1), 2-Pyrroloylglycine, Dityrosine) for the development of DM. The findings suggest that the combination of these potential metabolites may be used for the prognosis of pre-DM. Targeting the pathways that involved in these prognostic biomarkers would be beneficial for the regression to NGR and the early prevention of DM among pre-DM.

## Introduction

Pre-diabetes (pre-DM), typically defined as blood glucose levels above normal but below diabetes thresholds, has been increasing globally and has a high chance of developing diabetes mellitus (DM)^1^. It is estimated that there were 318 million adults suffering from impaired glucose tolerance in the world by 2015^2^. As to the Chinese adult population, a cross-sectional survey in 2010 reported that the estimated prevalence of pre-DM was 50.1%^3^. Previous studies^4^ have shown that about 5–10% of pre-DM progressed to DM every year, and persistent hyperglycemia leads to the complications that are the major source of morbidity, mortality, and cost. Nowadays, There is a common conception that this natural history is not inevitable^5^. Randomized controlled trials showed that lifestyle intervention or glucose-lowering medications could delay and even reverse the natural course of pre-DM^6–8^. The American Diabetes Association (ADA) recommends at least annual screening via testing fasting plasma glucose (FPG), 2-hr 75 g oral glucose tolerance test (OGTT), or hemoglobin A1c (HbA1c) in those with pre-DM^9^. However, clinicians are far from satisfied with the presently used method of monitoring glycemic level because of its hysteresis. FPG, OGTT and HbA1c are used more as a diagnostic than a predictive marker. Interestingly, multiple cross-sectional and prospective cohort studies have revealed the metabolism of impaired branched-chain amino acid (BCAA), aromatic amino acid (AAA), free fatty acid (FFA), acylcarnitines and glycerophospholipid are associated with insulin resistance, and many metabolites were considered as biomarkers for the prediction of pre-DM and DM^10–13^. However, little is known about the potential metabolic biomarkers of different glycemic prognoses among subjects with pre-DM. More importantly, building a metabolic model for predicting the transition from pre-DM to NGR or DM would be helpful for the early prevention and treatment among individuals with pre-DM.

Metabolomics provides a snapshot of the metabolic dynamics that reflects the response of living systems to pathophysiological stimuli and/or genetic modifications and surrounding environment. Furthermore, in many ways, tanscriptomic, genomic, and proteomic changes are upstream of the final physiology of cells, whereas the metabolic profile is likely closer in response to the disease process^14^. Ultra-performance liquid chromatography-quadrupole time-of-flight mass spectrometry (UPLC-QTOF-MS) of biofluids can easily detect hundreds of individual species in a single clinical sample, reflecting the biochemical fingerprint of the organism^15^. Characterized by sensitivity and high mass accuracy, the technique has been employed in identifying novel biomarkers for cancers^16,17^, metabolic disorders^18–20^, drug toxicity and function^21,22^, and so on.

With the current study, we characterized the metabolic profiles of fasting plasma samples among the 108 pre-DM at baseline with different outcomes ten years later utilizing untargeted UPLC-QTOF-MS analysis. 23 and 22 metabolites were identified as biomarkers for transition to NGR and DM from pre-DM, respectively. And the underlying biochemical pathways leading to different prognoses were investigated.

## Results

### Demographic and Clinical Characteristics

108 participants with pre-DM from a longitudinal cohort study were followed up for ten years and were divided into 3 groups according to different glycemic outcomes. 20 participants progressed to DM, 20 regressed to NGR, and 68 remained at pre-DM, respectively.

At baseline, there were no significant differences in ages, gender, body mass index, blood glucose, lipid profile, blood pressure as well as general health conditions among these 3 groups (Supplemental Table S1). At the end-point of the study, no significant differences in the biochemical characteristics were found among the three groups, except for fasting glucose, 2-h glucose and HbA1c (Table 1).

**Table 1:** Characteristics of the study participants at end-point.

	NGR (n = 20)	Pre-DM (n = 68)	DM (n = 20)
Age, years	57.4 ± 8.8	60.4 ± 8.9	57.9 ± 10.1
Gender, N, male/female	7/13	25/43	7/13
Body mass index, kg/m^2^	24.0 ± 3.1	24.8 ± 2.9	26.1 ± 3.9
Waist circumference, cm	83.3 ± 7.5	87.1 ± 8.4	88.3 ± 11.9
Waist-hip ratio	0.88 ± 0.04	0.90 ± 0.06	0.92 ± 0.08
Hypertension, %	45.0	57.4	70.0
Family history of DM, %	35.0	33.8	45.0
Current smoker, %	5.0	22.1	15.0
Current drinker, %	15.0	13.2	0.0
**Physical activity**			
Inactive, %	5.0	11.8	10.0
Medium, %	70.0	73.5	75.0
Active, %	25.0	14.7	15.0
SBP, mmHg	132.5 ± 19.4	138.9 ± 14.8	138.7 ± 17.5
DBP, mmHg	77.4 ± 8.8	80.5 ± 9.5	80.4 ± 10.4
Fasting glucose, mmol/L	5.2 ± 0.2^*^	5.6 ± 0.5	8.0 ± 2.9^**#^
2-h glucose, mmol/L	6.0 ± 0.8^**^	7.6 ± 1.8	13.1 ± 4.7^**#^
HbA1c, %	5.3 ± 0.5^**^	5.8 ± 0.3	7.2 ± 1.4^**#^
Fasting insulin, pmol/mL	8.4 ± 3.7	9.2 ± 5.0	11.3 ± 3.8^*a^
2-h insulin, pmol/mL	38.9 ± 23.5	71.2 ± 65.4	58.7 ± 36.8
HDL, mmol/L	1.4 ± 0.3	1.5 ± 0.4	1.5 ± 0.4
LDL, mmol/L	3.1 ± 0.7	3.0 ± 0.9	3.5 ± 1.1
TC, mmol/L	5.2 ± 1.2	5.5 ± 1.8	6.2 ± 1.3^b^
TG, mmol/L	1.4 ± 0.7	2.0 ± 2.6	2.8 ± 2.3^*#^
BUA, umol/L	311.9 ± 69.7	338.7 ± 88.5	321.3 ± 79.4
CR, umol/L	81.1 ± 18.6	72.8 ± 29.0	62.6 ± 25.7
ALT, U/L	20.8 ± 9.0	23.0 ± 16.8	33.1 ± 32.7
AST, U/L	24.5 ± 6.5	27.3 ± 17.8	28.1 ± 16.8
γGT, U/L	24.0 ± 21.4	31.8 ± 30.5	31.8 ± 17.2

Values are mean ± SD or %. *p < 0.01, **p < 0.001 compared to pre-DM and ^#^p < 0.001 compared to NGR. ^a^Exact significance (2-tailed), p = 0.015 compared to NGR; ^b^p = 0.017 compared to pre-DM. SBP: systolic blood pressure; DBP: diastolic blood pressure; HbA1c: hemoglobin A1c; HDL: high density lipoprotein; LDL: low density lipoprotein TC: total cholesterol; TG: triglyceride; BUA: blood uric acid; CR: creatinine; ALT: alanine transaminase; AST: aspartate transaminase; γGT: γ glutamyl transferase.

### Quality control

The robustness and stability of the method was assessed by repeat analysis of a representative pooled quality control (QC) sample during sample runs. The overlapped total ion current chromatograms of the QC sample demonstrated the repeatability of our UPLC-QTOF-MS system (Supplemental Fig. S1A). The principal component analysis (PCA) performed on QC and other groups revealed that QC samples were clustered in the PCA scores plot (Supplemental Fig. S2A). The percentage coefficient of variation (CV%) of peak intensity was estimated as 4.1–18.6%. These results collectively indicated good repeatability, reliability, and stability of this method for metabolite analysis.

### Plasma metabolite profile and makers for NGR and DM

**Figure 1:**
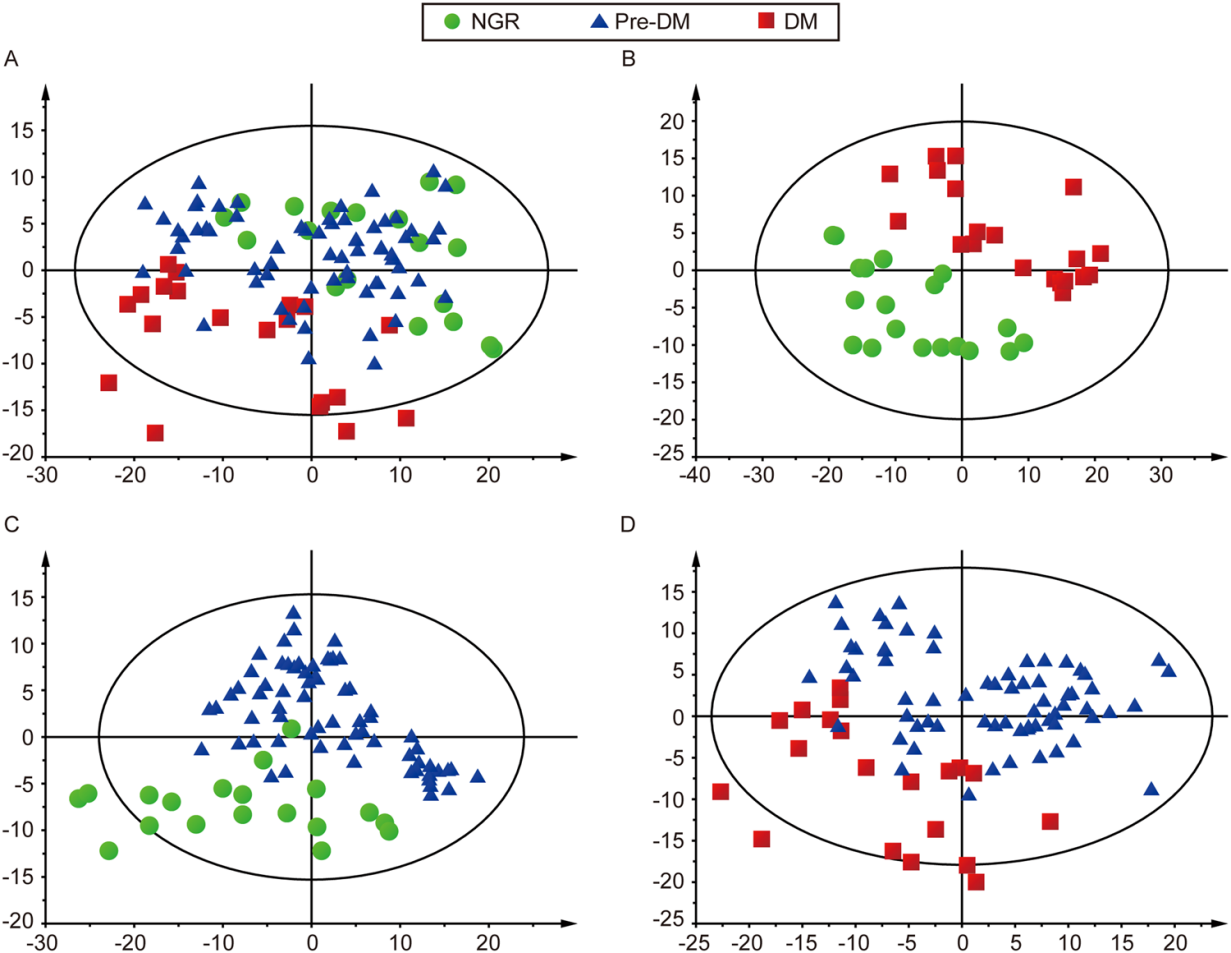
PLS-DA score plots of different groups based on plasma spectral data of UPLC-QTOF-MS positive ion mode. One point stands for one subject. (**A**) PLS-DA score plot of the NGR vs pre-DM vs DM groups. (**B**) PLS-DA score plot of the NGR vs DM groups. (**C**) PLS-DA score plot of the NGR vs Pre-DM groups. (**D**) PLS-DA score plot of the pre-DM vs DM groups.

Representative base peak intensity (BPI) chromatograms of plasma samples indicated that the sample metabolites attained suitable separation. Typical single UPLC-QTOF/MS base peak intensity chromatograms of a healthy control, a patient with pre-DM and a patient with DM are presented in Supplemental Fig. S1B. Multivariate statistical analysis was performed to determine whether the plasma metabolic profiles were different among participants progressed to DM, regressed to NGR and remained at pre-DM. The PCA score plots of the three groups, NGR vs DM, NGR vs pre-DM and pre-DM vs DM are presented in Supplemental Fig. S2B–D. The R2X values of PCA analysis were > 0.5 (0.651, 0.604, 0.631, respectively). Partial least squares discriminant analysis (PLS-DA) results of pair-wise groups indicated separations in the three groups (Fig. 1A) and sub-comparison groups (Fig. 1B–D) with valid model fits (R2Y(cum) > 0.7 and Q2(cum) > 0.4)^23^. Variable importance in projection (VIP) values were obtained from the models and pair-wise statistical test for difference was performed. Variables with VIP value > 1.0 and statistical p value < 0.05 were selected and verified by loading plots. Finally, a metabolite was annotated according to MS information, structure information, accurate mass, retention time, fragmentation pattern and standards.

Based on the above steps, a panel of 23 (Table 2) metabolites distinctively discriminated NGR and DM groups as well as NGR and pre-DM groups with a same trend, which were considered to be the potential metabolic biomarkers for the prognosis to NGR. Similarly, a panel of 22 (Table 3) metabolites discriminated NGR and DM groups as well as DM and pre-DM groups with a same trend were considered to be metabolic markers for the prognosis to DM. Compared to the pre-DM group, 18 biomarkers were up-regulated and 5 biomarkers were down-regulated in the NGR group, while 5 biomarkers increased and 17 biomarkers declined in the DM group. Of the substances that were screened in this study, several substances appear to be unknown. We will attempt to identify these unknown substances in future studies.

**Table 2:** Discriminative metabolites between NGR and pre-DM (ESI+ mode).

Metabolites	Measured mass, Da	Calculated mass, Da	Mass accuracy, ppm	Quasi-molecular ion	VIP^*^	FC^‡^	p^†^	Identification
2,3-Epoxymenaquinone	347.1612	347.1618	−1.7283	M + Na	1.36	1.50	0.0005	HMDB
Pc(14:1/16:1)	740.4650	740.4633	2.2959	M + K	1.52	1.32	0.0152	STD
5-methoxytryptamine	213.1025	213.1009	7.5081	M + Na	1.46	3.56	0.0041	STD
N(6)-(octanoyl)lysine	311.1732	311.1737	−1.6068	M + K	1.31	1.31	0.0204	STD
3-Phenylbutyric acid	356.1910	356.1906	1.1230	2 M + 3H2O + 2 H	1.31	1.29	0.0337	HMDB
Lysyl-Tyrosine	327.2046	327.2028	5.5011	M + NH4	1.29	1.22	0.0401	HMDB
N6-Acetyl-L-lysine	399.2205	399.2214	−2.2544	2 M + Na	1.30	1.23	0.0468	STD
Pantetheine	301.1185	301.1198	−4.3172	M + Na	1.35	1.46	0.0013	HMDB
S-(hydroxymethyl)glutathione	355.1262	355.1287	−7.0398	M + NH4	1.28	1.52	0.0042	STD
3-Ethylphenol	283.1110	283.1095	5.2983	2 M + K	1.11	1.34	0.0085	HMDB
LysoPE(20:5/0:0)	532.2992	532.3034	−7.8490	M + CH3OH + H	1.26	1.18	0.0437	HMDB
Delta 8,14 -Sterol	487.2746	487.2739	1.4366	M + + 2 K + H	1.26	1.23	0.0488	HMDB
Caprylic acid	306.2666	306.2639	8.8158	M + NH4	1.08	1.15	0.0319	HMDB
1-Stearoylglycerophosphoglycerol	576.3257	576.3272	−2.5767	M + ACN + Na	1.33	1.63	0.0028	HMDB
Endomorphin-1	649.2475	649.2535	−9.2415	M + K	1.57	1.73	0.0026	HMDB
20-Hydroxy-leukotriene E4	473.2654	473.2680	−5.4937	M + NH4	1.28	1.28	0.0004	HMDB
Lysopc(18:3)	518.3237	518.3241	−0.8007	M + H	1.86	0.29	0.0020	STD
Lysopc(20:5)	542.3238	542.3241	−0.5808	M + H	1.73	0.11	0.0000	STD
cis-13,16-Docosadienoic acid	354.3349	354.3372	−6.4910	M + NH4	1.54	1.60	0.0029	HMDB
Lysopc(20:4)	544.3356	544.3398	−7.6515	M + H	1.60	0.70	0.0397	STD
L-palmitoylcarnitine	400.3410	400.3421	−2.8351	M + H	2.11	2.25	0.0075	STD
Pc(18:3/20:3)	806.5683	806.5694	−1.4022	M + H	1.09	0.38	0.0336	HMDB
LysoPC(20:3)	546.3487	546.3554	−12.2925	M + H	1.62	0.14	0.0013	STD

^*^Variable importance in the projection (VIP) was obtained from PLS-DA with a threshold of 1.0. ^†^p values were calculated from tests of statistical difference. Difference was considered statistically significant when p < 0.05. ^‡^Fold change (FC) was calculated from the arithmetic mean values of NGR and pre-DM groups. Fold change with a positive value indicates a relatively higher concentration present in NGR patients while negative indicates lower.

**Table 3:** Discriminative metabolites between DM and pre-DM (ESI+ mode).

Metabolites	Measured mass, Da	Calculated mass, Da	Mass accuracy, ppm	Quasi-molecular ion	VIP^*^	FC^‡^	p^†^	Identifi-cation
Pc(16:0/14:0)	782.4430	782.4499	−8.8121	M + 2 K + H	1.16	0.82	0.0351	HMDB
2-Pyrroloylglycine	191.0420	191.0427	−3.7165	M + Na	1.19	0.84	0.0070	STD
Dityrosine	383.1181	383.1219	−9.9186	M + Na	2.43	1.23	0.0188	STD
Kynuramine	203.0561	203.0581	−9.9529	M + K	3.11	1.25	0.0007	HMDB
L-lysine	188.1400	188.1394	3.4496	M + ACN + H	1.27	0.74	0.0025	STD
L-threonine	164.0296	164.0294	1.2010	M + 2Na-H	2.33	0.59	0.0002	STD
5-hydroxy-2-oxo-4-ureido-2,5-dihydro-1h-imidazole-5-carboxylate	220.0656	220.0676	−9.2791	M + NH4	1.85	0.69	0.0002	HMDB
1,3,7-trimethyluric acid	274.0925	274.0911	5.2719	M + ACN + Na	1.98	1.52	0.0071	STD
Betaine	118.0858	118.0863	−3.8531	M + H	1.28	0.71	0.0006	HMDB
Iso-valeraldehyde	104.1077	104.1075	1.9211	M + NH4	2.16	0.74	0.0000	HMDB
L-carnitine	162.1132	162.1130	1.2337	M + H	1.19	0.73	0.0005	STD
2-ketobutyric acid	103.0397	103.0395	1.9410	M + H	1.39	0.72	0.0003	HMDB
3,5-dihydroxybenzoic acid	187.0605	187.0601	2.1490	M + CH3OH + H	1.77	0.61	0.0010	HMDB
Uric acid	169.0358	169.0356	1.0885	M + H	1.97	0.20	0.0001	STD
Lysope(16:0/0:0)	454.2908	454.2928	−4.4355	M + H	2.07	0.24	0.0006	HMDB
Pantetheine	301.1181	301.1198	−5.6456	M + Na	1.15	0.74	0.0336	HMDB
Palmitic amide	256.2629	256.2635	−2.3062	M + H	1.42	0.41	0.0279	HMDB
3-dehydroxycarnitine	184.0736	184.0740	−2.1730	M + K	1.63	1.30	0.0061	STD
Lysopc(18:1)	544.3363	544.3403	−7.3484	M + H	1.06	0.71	0.0184	STD
Linoleic acid	341.3053	341.3050	0.8174	M + H	1.14	1.19	0.0212	HMDB
Lysopc(18:0)	524.3706	524.3711	−0.8887	M + H	1.34	0.42	0.0398	STD
Pc(18:0/18:2)	786.5968	786.6007	−4.9975	M + H	1.29	0.09	0.0054	HMDB

^*^Variable importance in the projection (VIP) was obtained from PLS-DA with a threshold of 1.0. ^†^p values were calculated from tests of statistical difference. Difference was considered statistically significant when p < 0.05. ^‡^Fold change (FC) was calculated from the arithmetic mean values of DM and pre-DM groups. Fold change with a positive value indicates a relatively higher concentration present in DM patients while negative indicates lower.

### Metabolic pathways

**Figure 2:**
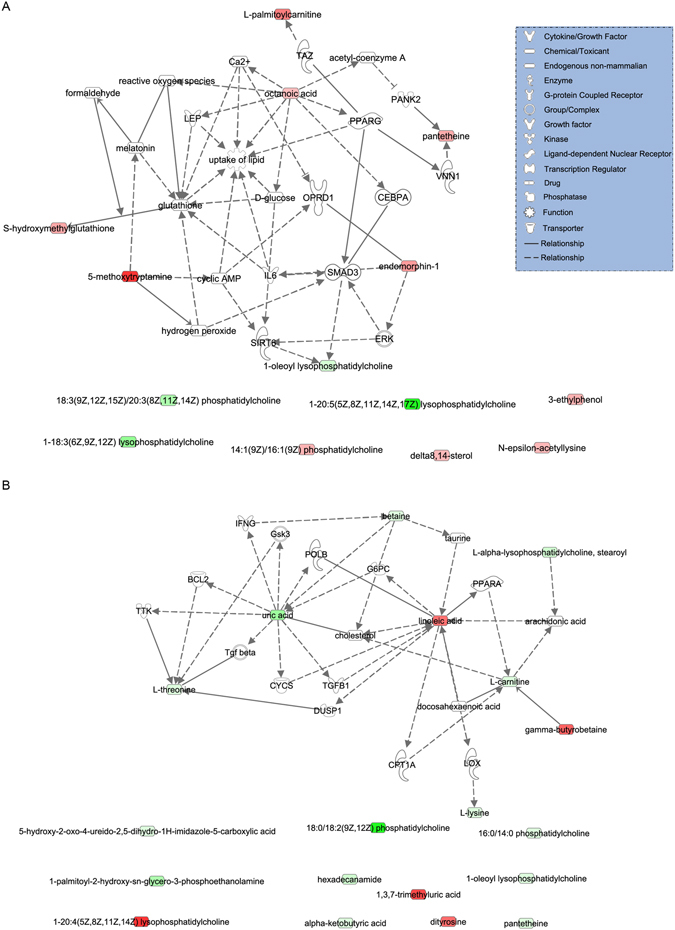
Biological network and canonical pathways related to the identified metabolites in NGR (**A**) and DM (**B**). Molecules are represented as nodes, and the biological relationship between two nodes is represented as a line. Red symbols represent up-regulated metabolites; green symbols represent down-regulated metabolites. The solid lines and dotted lines show direct and indirect functional relationships, respectively.

Pathway analysis carried out by IPA software revealed that three metabolic pathways including glycerophospholipid metabolism, Lipoate Biosynthesis and Incorporation II, and Melatonin Degradation II were found to contribute in the process from pre-DM to NGR (Fig. 2A). While L-carnitine Biosynthesis, Superpathway of Methionine Degradation, Mitochondrial L-carnitine Shuttle Pathway, and Choline Degradation I were associated with the development of DM (Fig. 2B).

### Verification and Optimization of Potential Biomakers

Receiver operating characteristic (ROC) curves for each potential biomarker were established to test the probability of ‘single biomarkers’. Area under curve (AUCs) and their CV% values in QC samples are showed in Supplemental Tables S2 and S3. To assess how multiple metabolites collectively classify the NGR/DM and the Pre-DM groups, we built logistic regression model using stepwise selection on the 23/22 metabolites for the samples, respectively.

**Figure 3:**
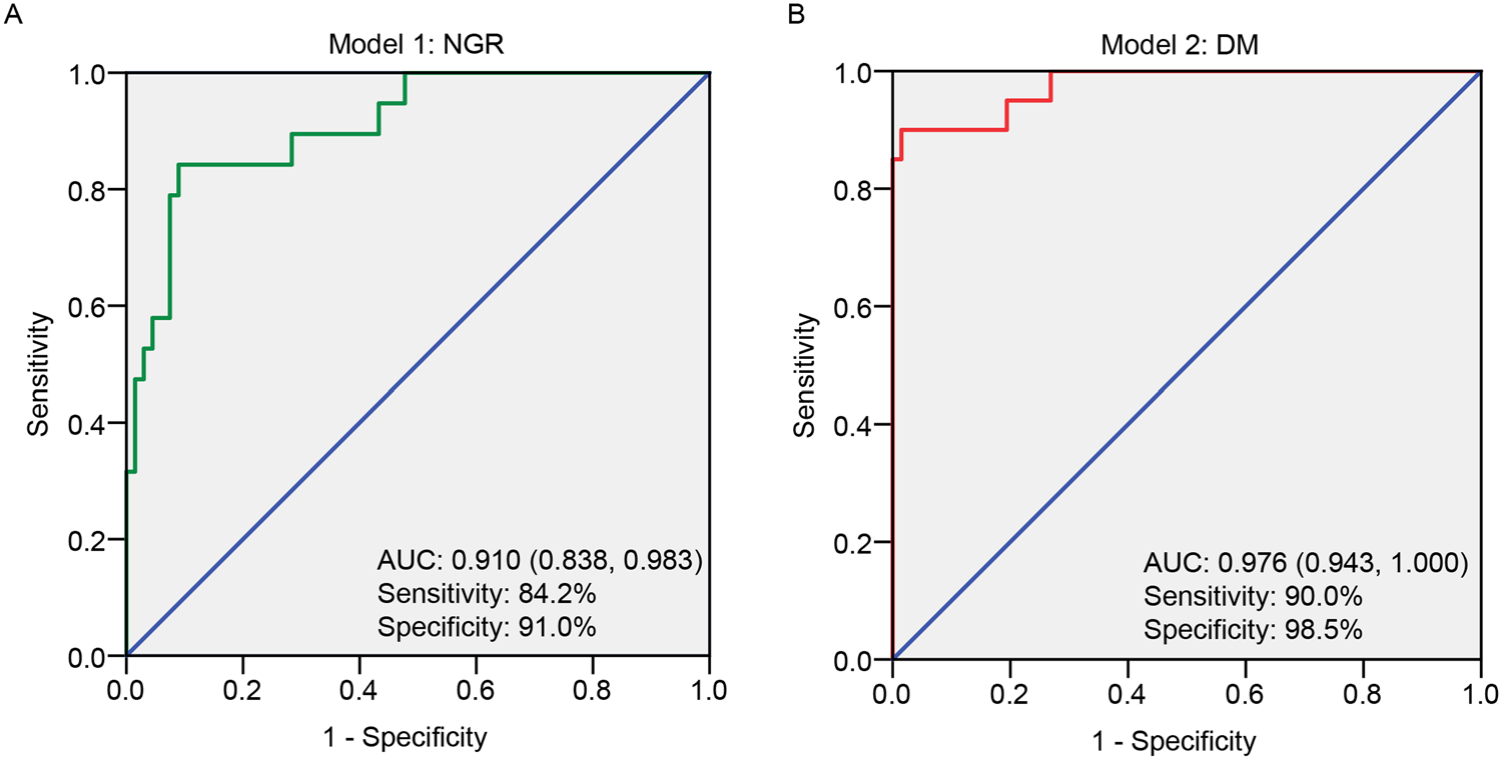
(**A**) ROC analysis for discrimination of pre-DM and NGR groups by logistic regression model combining 20-Hydroxy-leukotriene E4, Lysopc(20:4), 5-methoxytryptamine, Endomorphin-1, Lysopc(20:3). (**B**) ROC analysis for discrimination of pre-DM and DM groups by logistic regression model combining Iso-valeraldehyde, linoleic acid, Lysopc(18:1), 2-Pyrroloylglycine, Dityrosine.

From pre-DM to NGR, five metabolites entered the multiple regression model: 20-Hydroxy-leukotriene E4 (standardized [std] β = 2.135, p = 0.004), Lysopc(20:4) (std β = −1.423, p = 0.027), 5-methoxytryptamine (std β = 0.606, p = 0.047), Endomorphin-1 (std β = 4.685, p = 0.018),and Lysopc(20:3) (std β = −19.176, p = 0.002) with an overall correct percentage of 86.0%. We calculated the sensitivity and specificity based on estimates of the final model built on the samples (logitP1 = −1.847 + 2.135(20-Hydroxy-leukotriene E4) − 1.423(Lysopc(20:4) + 0.606 (5-methoxytryptamine) + 4.685 (Endomorphin-1) − 19.176 (Lysopc(20:3)), and the model fit very well (AUC = 0.910, 95% CI [0.838, 0.983], sensitivity = 84.2%, specificity = 91.0%) with cut-off value −0.08 and p value < 0.001.

Meanwhile, five metabolites entered the multiple regression model from pre-DM to DM: Iso-valeraldehyde (standardized [std] β = −1.544, p = 0.049), linoleic acid (std β = 2.194, p = 0.019), Lysopc(18:1) (std β = −4.769, p = 0.013), 2-Pyrroloylglycine (std β = −2.922, p = 0.044) and Dityrosine (std β = 0.285, p = 0.007) with an overall correct percentage of 95.4%. The final model (logitP2 = −1.119–5.144(Iso-valeraldehyde) + 2.194(linoleic acid) − 4.769(Lysopc(18:1) − 2.922(2-Pyrroloylglycine) + 0.285 (Dityrosine)) showed satisfactory fitness (AUC = 0.976, 95% CI [0.943, 1.000], sensitivity = 90.0%, specificity = 98.5%) with cut-off value −0.33 and p value < 0.001. The ROC curves of the combined biomarkers are shown in Fig. 3. Both of them were statistically different from single metabolites as they showed a higher lower bound of 95% CI of AUCs than most of the upper bounds of 95% CI of single metabolites, and were further validated by comparison of AUCs with MedCalc Statistical Software with a statistical p value < 0.05^24^.

**Figure 4:**
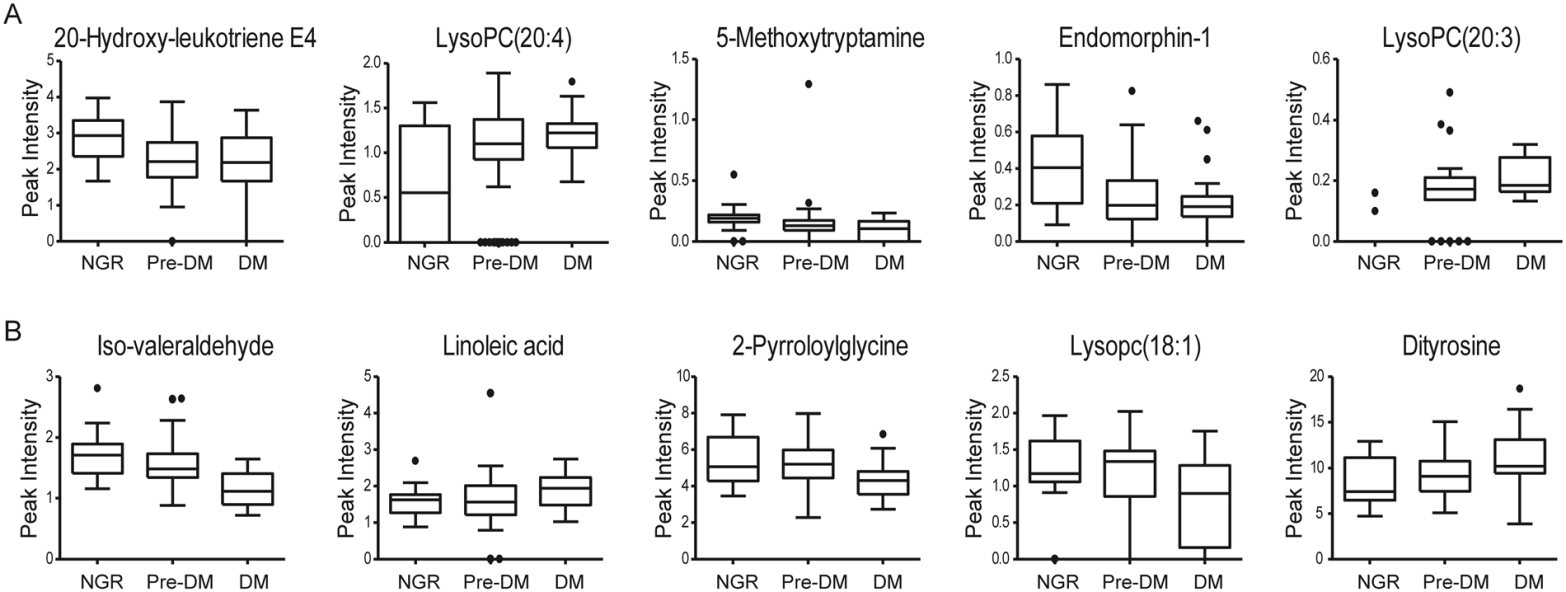
Box plots of mean intensity of ten representative metabolites in plasma samples of NGR, pre-DM and DM patients.

Relative concentrations of these metabolites in the logistic regression equations are presented in Fig. 4. These data strongly support the robustness of UPLC-QTOF-MS to identify metabolic differences in the plasma samples of pre-DM patients with different prognoses (NGR or DM).

## Discussion

Early diagnosis in hyperglycemia, prior to developing into diabetes, can improve the living quality of those suffering from pre-DM. The rapid rise in pre-DM worldwide raises urgent needs to develop effective prognostic biomarkers and prognosis evaluation methods based on the easily accessible materials, such as blood and urine.

Using UPLC-QTOF-MS based metabolic profiling combined with pattern recognition techniques on plasma samples, we identified molecular markers that discriminate the prognoses to NGR and to DM in 108 pre-diabetic patients in a longitudinal study with more than ten years of follow-up. In this study, a total of 23 metabolites involved in different biochemical metabolic pathways with high statistical significance were associated with the outcome of NGR, while 22 metabolites with the outcome of DM.

Of these perturbed metabolic pathways, particular importance should be given to glycerophospholipid metabolism. Earlier studies have demonstrated that diabetes is intimately associated with metabolic disorders of lipids, especially phospholipids^25,26^. Phosphocholine metabolites, including PC and LysoPC, are key components of the biomembranes of cells, as well as participating in various biological pathways^27,28^, especially in cellular signaling and metabolism. Correspondingly, significant changes in phosphatidylcholine metabolism were observed in our study. PC (16:0/14:0) and PC (18:0/18:2) showed a reduction in level from pre-DM to DM, while PC (14:1/16:1) increased and PC (18:3/20:3) decreased from pre-DM to NGR. This is in line with a previous prospective cohort study based on 866 participants with seven years’ follow-up that a series of PC such as C32:1, C36:1, C38:3, and C40:5 were independently associated with increased risk of T2D and C34:3, C40:6, C42:5, C44:4, and C44:5 with decreased risk^29^. However, it was also demonstrated that the type of linkage between phospholipid core and fatty acid residue may be the key factor contributing to the antithetical association between two phosphatidylcholine subclasses and T2DM risk^29^. Therefore, the specific structural formula of the PCs detected needs further validation.

What’s more, some studies have reported that changed concentrations of lysoPCs are associated with the risk of T2DM, especially lysoPC(18:2), which was significantly altered in patients with impaired glucose tolerance (IGT) and was identified as an IGT-specific biomarker^30^. LysoPCs can mediate many cell-signaling pathways in monocytes/macrophages^29,31^ and specific receptors^32^, and therefore participate in the inflammatory response. In our results, several lysoPC species (LPC C20:4, LPC C18:3, LPC C20:5, LPC C20:3) were down-regulated from pre-DM to NGR group, while the plasma level of LPC C18:1 were higher and LPC C18:0 were lower in DM than pre-DM patients, possibly owing to altered activity of phospholipase A2 which catalyzes PC hydrolysis to lysoPC^33^. In mouse models, Yea, K *et al*. have reported that the blood glucose lowering effect of LPC were found to be sensitive to variations in LPC acyl chain length^34^, which may elucidate the divisive findings mentioned above.

In recent years, 5-methoxytryptamine (5-MT) has been revealed to play a pivotal role in the alternative melatonin synthetic pathway, in which serotonin is first O-methylated to 5-MT and, thereafter, 5-MT is N-acetylated to melatonin (N-acetyl-5-methoxytryptamine)^35^. Melatonin was reported as a free radical scavenger and an antioxidant for protection from the oxidative stress^36–38^, especially reducing oxidative damage to lipids, which is important for the maintenance of mitochondrial homeostasis. Letra-Vilela, R. *et al*. observed that removal of the N-acetyl group enhances the antioxidant and neuroprotective properties of the maletonin^39^. The elevated level of plasma 5-MT concentrations contributed to scavenge radicals and radical products and functioned as antioxidant against reaction oxygen species (ROS). Meanwhile, an increased level of plasma S-(hydroxymethyl)glutathione concentration was observed in patients regressed to NGR. S-(hydroxymethyl)glutathione interconverts with glutathione (GSH), later of which is a key antioxidant and marker for conditions with oxidative stress^40^. Therefore, we can conclude that a balance between oxidation and antioxidation was achieved in patients regressed to NGR.

Caprylic acid, also named octanoic acid, is a medium-chain fatty acid which down-regulates a number of key adipogenic genes including peroxisome proliferator activated receptor (PPAR), CCAAT/enhancer binding protein alpha. Rats fed on diet rich in medium-chain fatty acids had smaller fat pads, reduced adipose tissue lipoprotein lipase activity and improved insulin sensitivity and glucose tolerance^41^. Thus the elevated level of caprylic acid may be another contributor to glucose tolerance recovery.

In this study, a major alteration observed in patients progressed to DM was amino acid metabolism with a decreased level of L-lysine, L-threonine, Betaine, Iso-valeraldehyde, 2-ketobutyric acid, and 2-Pyrroloylglycine. 2-Ketobutyric acid is a substance that is involved in the metabolism of many amino acids, such as glycine, methionine, valine, leucine, serine, threonine and isoleucine. It is also one of the degradation products of threonine, which can be converted to propionyl-CoA (and subsequently methylmalonyl CoA, which can be converted to succinyl CoA, a citric acid cycle intermediate), and thus enter the citric acid cycle. Our research is consistent with the report that 2-ketobutyric acid is associated with both prevalent diabetes (OR: 1.43, 95% CI (1.06,1.92)) and incident diabetes (OR: 1.81, 95% CI (1.35, 2.42)^42^ while glycine is found inversely associated with diabetes risk^43,44^. This suggest that increased activity in the L-threonine-2-ketobutyric acid-citric acid cycle and decreased glycine, which plays an important role in metabolic regulation and anti-oxidative reactions^45^ may account for diabetes risk.

Betaine, also glycine betaine, has been reported to be disturbed in diabetes and it is regarded as a marker of diabetes in plasma and urine samples^46–48^. It is inversely associated with several components of metabolic syndrome including obesity, hypertension, and hyperlipemia^49^. Mouse model studies suggest increased betaine metabolism in diabetes, which could be expected to lower plasma betaine^50^. In agreement with these findings, a reduction of betaine level is an important contribution to DM development.

A decreased level of L-carnitine and an increased level of dityrosine and 3-dehydroxycarnitine were detected in patients progressed to DM. These results are consistent with an interesting study investigating the relationship between the consumption of red meat and the development of associated diseases^51^. L-carnitine is known to be a long-chain fatty acid transporter in the “Mitocondrial L-carnitine Shuttle Pathway”^52^, and 3-dehydroxycarnitine has been identified as an intermediate metabolite in the intestinal bacterial catabolism of L-carnitine^53^, which may give a hint the involvement of gut microbiota in DM development. Dityrosine has been proposed as a biomarker of oxidative stress under a variety of conditions and biological systems including aging, exposure to oxygen free radicals, nitrogen dioxide, and lipid hydroperoxides^54^. Increases in dityrosine levels have been associated with pathologies such as atherosclerosis, Alzheimer’s Disease, and so on^55,56^. Together, these findings reflect impaired mitochondrial β-oxidation and perturbed fatty acid metabolism in the development of insulin resistance.

To make it better to apply the identified biomarkers for prediction of pre-DM prognosis in the long run in clinical setting, the key of this study was to select and combine several specific biomarkers for establishing a noninvasive and accurate predict method for prognosis of pre-DM. The candidate biomarker selection rationale was as follows: first, the biomarkers must be confirmed by standards; second, the biomarkers with high VIPs in the pattern recognition analysis and significant discrepancy between groups; and last, the biomarkers in the logistic regression equation with higher AUCs and predictive sensibility and specificity. As a result, five biomarkers (20-Hydroxy-leukotriene E4, Lysopc(20:4), 5-methoxytryptamine, Endomorphin-1, Lysopc(20:3)) and five biomarkers (Iso-valeraldehyde, linoleic acid, Lysopc(18:1), 2-Pyrroloylglycine, Dityrosine) were included in the predictive equation of NGR and DM, respectively.

In summary, our study comprehensively captured alterations in the human metabolome associated with different glucose tolerance outcomes of pre-DM in a longitudinal cohort study. A decreased glycerophosphoslipid metabolism and balanced oxidation and antioxidation contributed to the transition to NGR, while impaired amino acid metabolism and perturbed mitochondrial β-oxidation were associated with the development of DM. Targeting the pathways that involve in these newly prognosis biomarkers would be beneficial for the regression to NGR and the early prevention of DM among participants with pre-DM.

## Subjects and Methods

### Ethics Statement

This study was approved by Ruijin Hospital Ethics Committee (approval no. 2014–114).

Written informed consent signed by each of participants was provided before blood samples were taken. All methods were carried out in accordance with the relevant guidelines and regulations.

### Study design and Subjects

This study was from a population-based prospective cohort study of 2132 men and women aged 18–76 years, from November 2002 to January 2003, among whom 778 participants were pre-DM at baseline. The follow-up visit was conducted from July 2013 to October 2014 and 526 participants who were pre-DM at baseline were followed, among whom 334 individuals both answered questionnaires and had plasma glucose measurement during an oral glucose tolerance test (OGTT). Serum lipid profile and liver function were also assayed. After excluded the individuals on anti-diabetes medication or with serious liver, renal dysfunction and cancer, the remaining 108 individuals were included in our final analysis. This study design has been described previously^57–59^.

According to different glycemic outcomes at follow up, the 108 participants were divided into 3 groups. 20 participants progressed to diabetes DM, 20 regressed to NGR, and 68 remained at pre-DM. Details of the study population are presented in Supplemental Fig. S3.

Venous blood samples were collected at baseline and follow-up. The glucose level was measured by means of glucose oxidase method. Pre-DM and diabetes were diagnosed according to American Diabetes Association (ADA) 2010 Guidelines^60^. Pre-DM refers to subjects with impaired fasting glucose (fasting glucose ranging from 5.6 to < 7.0 mmol/L, as well as 2-hour glucose < 7.8 mmol/L) and subjects with impaired glucose tolerance (2-hour glucose ranging from 7.8 to < 11.1 mmol/L). Both impaired fasting glucose and impaired glucose tolerance were confined to non-diabetic fasting and 2-hour concentrations. Fasting plasma samples were used to analyze biochemical indexes and metabolomics.

### Sample collection and preparation

Fasting blood samples were drawn under sterile conditions from an antecubital vein of all the study participants between 6:30 and 9:30 after a 12-hour overnight fast, and were collected directly into heparinized tubes. The tubes were centrifuged at 12000 g for 10 min and the supernatant (plasma sample) was aspirated and stored at −80 °C until analysis.

The plasma sample (100 μl) was thawed at 4 °C. 100 μl of plasma was spiked with 300 μl mixed solution (methanol: acetonitrile = 3:2) and vigorously vortexed for 30 seconds. The sample solution was centrifuged at 12000 × *g* for 10 min at 4 °C, and the supernatant was analyzed using UPLC-QTOF-MS. Study samples were analyzed in random order using a random-number generator in Excel 2015 (Microsoft, Redmond, Washington). QC samples were prepared by mixing equal volumes (10 μl) of different individual plasma samples and one QC sample was run after every ten study sample injections throughout the analytical workflow.

### UPLC-QTOF-MS conditions

In this study, a Waters ACQUITY^TM^ ultra performance liquid chromatography system (Waters Corp., Milford, USA) coupled with aSynaptG2 quadrupole time-of-flight (Q/TOF) tandem mass spectrometer (Waters, Milford, MA) was used to perform the analysis of plasma samples. The set-up parameters for the UPLC-QTOF-MS analysis were as follows: A T3 C18 chromatographic column (Waters, 2.1 mm × 100 mm, 1.7 μm) was used to separate metabolites contained in plasma with column temperature set at 45 °C. The eluted solution was 0.1% formic acid combined with 5 mM ammonium acetate in water (A) and acetonitrile (B) with a flow rate of 300 μl/min. The gradient elution program for analysis of plasma samples was as follows: 0–1 min, A: 98%; 1–3 min, A: 98–50%; 3–8 min, A: 50–45%; 8–12 min, A: 45%; 12–17 min, A: 45%−10%; 17–20 min, A: 10–98%.

The MS parameters were set up as follows: the electrospray ionization source (ESI) interface operated with a positive mode, capillary voltage of 3000 V, sample cone voltage of 40 V, extraction cone voltage of 4.0 V, desolvation gas flow of 650 L/h at 450 °C, source temperature of 120 °C, and cone gas flow of 50 L/h. Centroid data were collected under a scan time of 0.25 s and an inter scan delay of 0.02 s condition in continuum mode which ranged from m/z 100 to m/z 1200 Da.

To avoid possible contamination and keep the signal stable, the Q-TOF mass spectrometer system was tuned for optimum accuracy and reproducibility using leucine-enkephalin (m/z 556.2771) as the lock mass in all analyses at a concentration of 0.5 μg/mL. The lock spray frequency was set at 5 s and the lock mass data were averaged over 10 scans. MS^E^ was applied for the MS2 analysis with the low collision energy of 5 eV and the high collision energy of 30 eV.

### Data extraction and multivariate statistics

The raw data produced by UPLC-QTOF-MS were initially processed using MarkerLynx Applications Manager version 4.1 (Waters Corp., Manchester, UK). The data were peak-detected and noise-reduced so that only true peaks are further processed by the software. The data were presented with the ion intensities corresponding the retention time and m/z for each peak. The main parameters were set as follows: retention time window 0.5–16.5 min, mass range 100–1200 Da, XIC window 0.02 min, automatically calculate peak width and peak-peak baseline noise, use the raw data during the deconvolution procedure, marker intensity threshold (count) 1000, mass window 0.02 Da, retention time windows 0.2 min, noise elimination level 6.0, and retain the isotopic peaks. The internal standard was used for data quality control and data normalization (reproducibility). The ion peaks generated by the internal standard were removed and the metabolites were filtered by the QC samples.

The 80% rule was applied to treat the missing values^61^ and a data matrix that consisted of the ion intensities corresponding the retention time and m/z for each peak was generated and then exported to Simca-P software (v13.0, Umetrics, Umea, Sweden) followed by a series of pattern recognition (PR) methods. Multivariate statistical analyses, including principal component analysis (PCA) and a partial least squares discriminant analysis (PLS-DA) were carried out using SIMCA-P 13.0 software. The score plots from PLS-DA showed the differentiation of metabolic profiles of different groups. In addition, loading plots indicated the variables contributing to the classification. The quality of the model was described by the cross-validation parameter Q2 (cum), and R2Y, which represents the total explained variation for the X matrix. After the analysis of the three groups, pair-wise analysis (NGR vs pre-DM, DM vs pre-DM and NGR vs DM) was conducted to searching for the discrepant metabolites between groups.

### Identification of potential biomarkers and metabolic pathway analysis

The metabolites responsible for the separation of metabolic profiles of the pair-wise groups were obtained based on a variable importance in projection (VIP) threshold (VIP > 1 represented higher influence on the classification)^17^ from PLS-DA models accompanied with loading plots and a statistical test for difference (*p* < 0.05 was considered significant). A two-tailed t test or a nonparametric Mann-Whitney test was used for significance evaluation following data normality test with Shapiro-Wilk tests, which was performed with the R statistical software 3.3.2 for Windows.

A metabolite was detected and identified based on accurate mass, retention time, MS information and metabolite structure information from related databases: METLIN (https://metlin.scripps.edu/index.php) and HMDB (http://www.hmdb.ca/). Some of the metabolites were confirmed by comparison of retention time and fragmentation pattern with authentic standards.

The pathway analysis and network of potential biomarkers contributing to the classification between groups was carried out by IPA software (IPA, Ingenuity^s^ Systems, http://www.ingenuity.com).

**Figure 5:**
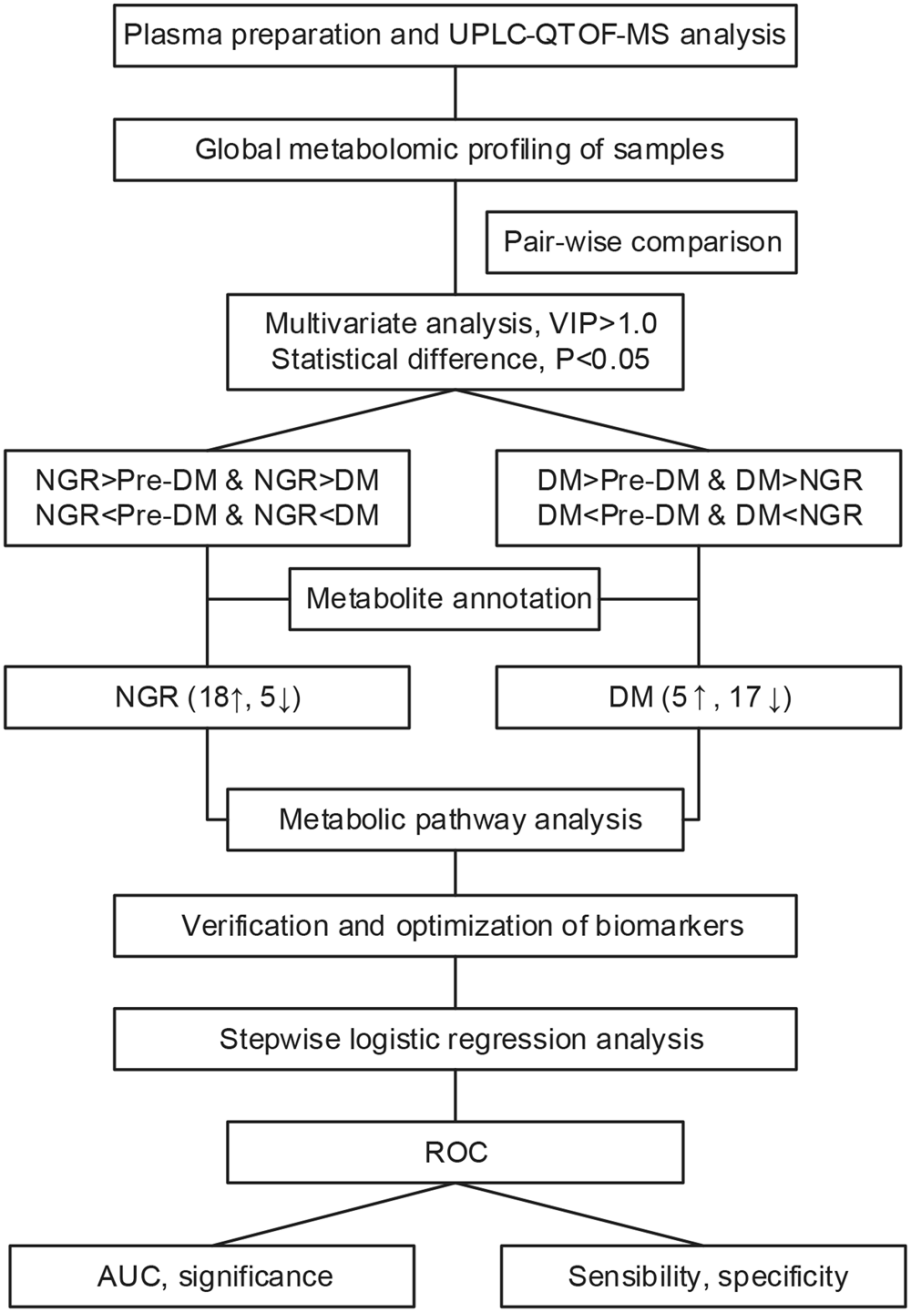
Schematic flow chart of the metabolomics analysis in the study. NGR: normal glucose regulation; Pre-DM: pre-diabetes; DM: diabetes; UPLC-QTOF-MS: Ultra-performance liquid chromatography-quadrupole time-of-flight mass spectrometry; VIP: variable importance in the projection; ROC: receiver operating characteristic curve; AUC: area under the curve.

The schematic flow chart of the metabolic profiling and biomarker identification and optimization strategy used in the study is shown in Fig. 5.

### Statistical analysis

Demographic and biochemical characteristics were presented as the mean (±SD) for continuous variables and % for categorical variables. Data normality and homogeneity of continuous variables were confirmed with Shapiro-Wilk tests and Levene tests, respectively. If data were normally distributed and variances were equal, data were analyzed by means of one-way analysis of variance (ANOVA) with LSD test; Otherwise, Kruskal-Wallis test with a nonparametric two-tailed Mann-Whitney test was used (alpha was adjusted to 0.05/3 here). And categorical variables were analyzed by χ2 test or Fisher’s exact test. Difference was considered statistically significant when p < 0.05.

Multiple logistic regression analysis of the potential metabolites was performed and receiver operating characteristics (ROC) analysis was used to evaluate predictive ability of potential metabolic biomarkers. Area under the curve (AUC), best cut-off point, sensitivity and specificity were determined using the maximum value of the Youden index. The analyses were performed using SPSS software version 23.0 (IBM Corp., USA).

## Electronic supplementary material

Supplementary information
