# Author Correction: Metabolic Biomarkers for Prognostic Prediction of Pre-diabetes: results from a longitudinal cohort study

**DOI:** 10.1038/s41598-017-14856-1

**Published:** 2017-11-21

**Authors:** Hailuan Zeng, Renchao Tong, Wenxin Tong, Qiaoling Yang, Miaoyan Qiu, Aizhen Xiong, Siming Sun, Lili Ding, Hongli Zhang, Li Yang, Jingyan Tian

**Affiliations:** 1 Department of Endocrinology and Metabolism, Shanghai Institute of Endocrine and Metabolic Diseases, Ruijin Hospital, Shanghai Jiao Tong University School of Medicine, Shanghai, China; 2 The MOE Key Laboratory for Standardization of Chinese Medicines, Institute of Chinese Materia Medica, Shanghai University of Traditional Chinese Medicine, Shanghai, China; 3 Department of Diabetes Complications & Metabolism, Beckman Research Institute, City of Hope, 1500 East Duarte Road, Duarte, CA USA; 4 Center for Chinese Medical Therapy and Systems Biology, Shanghai University of Traditional Chinese Medicine, Shanghai, China

*Scientific Reports* **7**:6575; doi:10.1038/s41598-017-06309-6; Article published online 26 July 2017

In the original version of this Article, Hailuan Zeng and Renchao Tong were omitted as equally contributing authors. This has now been corrected in the PDF and HTML versions of the paper.

