# Supplementary material for: Metabolic Biomarkers for Prognostic Prediction of Pre-diabetes: results from a longitudinal cohort study
Source: Sci Rep. 2017 Jul 26;7:6575. doi: 10.1038/s41598-017-06309-6 (PMC5529507; doi:10.1038/s41598-017-06309-6)
Supplement: Supplementary file 1 — Supplementary information [file 41598_2017_6309_MOESM1_ESM.pdf]

Supplementary information

Metabolic Biomarkers for Prognostic Prediction of Pre-diabetic Patients: results from a longitudinal cohort study

Hailuan Zeng[1], Renchao Tong[1], Wenxin Tong[1], Qiaoling Yang[1,2], Miaoyan Qiu[1], Aizhen Xiong[1], Siming Sun[2], Lili Ding[1,2], Hongli Zhang[1,2], Li Yang[1,*], Jingyan Tian[1,2,*]

[1]Department of Endocrinology and Metabolism, Shanghai Institute of Endocrine and Metabolic Diseases, Ruijin Hospital, Shanghai Jiao Tong University School of Medicine, Shanghai, China; The MOE Key Laboratory for Standardization of Chinese Medicines, Center for Chinese Medical Therapy and Systems Biology, Shanghai University of Traditional Chinese Medicine, Shanghai, China

[2]Department of Diabetes Complications & Metabolism, Beckman Research Institute, City of Hope, 1500 East Duarte Road, Duarte, CA

Hailuan Zeng and Renchao Tong contributed equally to this work. Correspondence and requests for materials should be addressed to J. T. (tianjypaper@163.com) or Y. L. (yl7@shutcm.edu.cn).

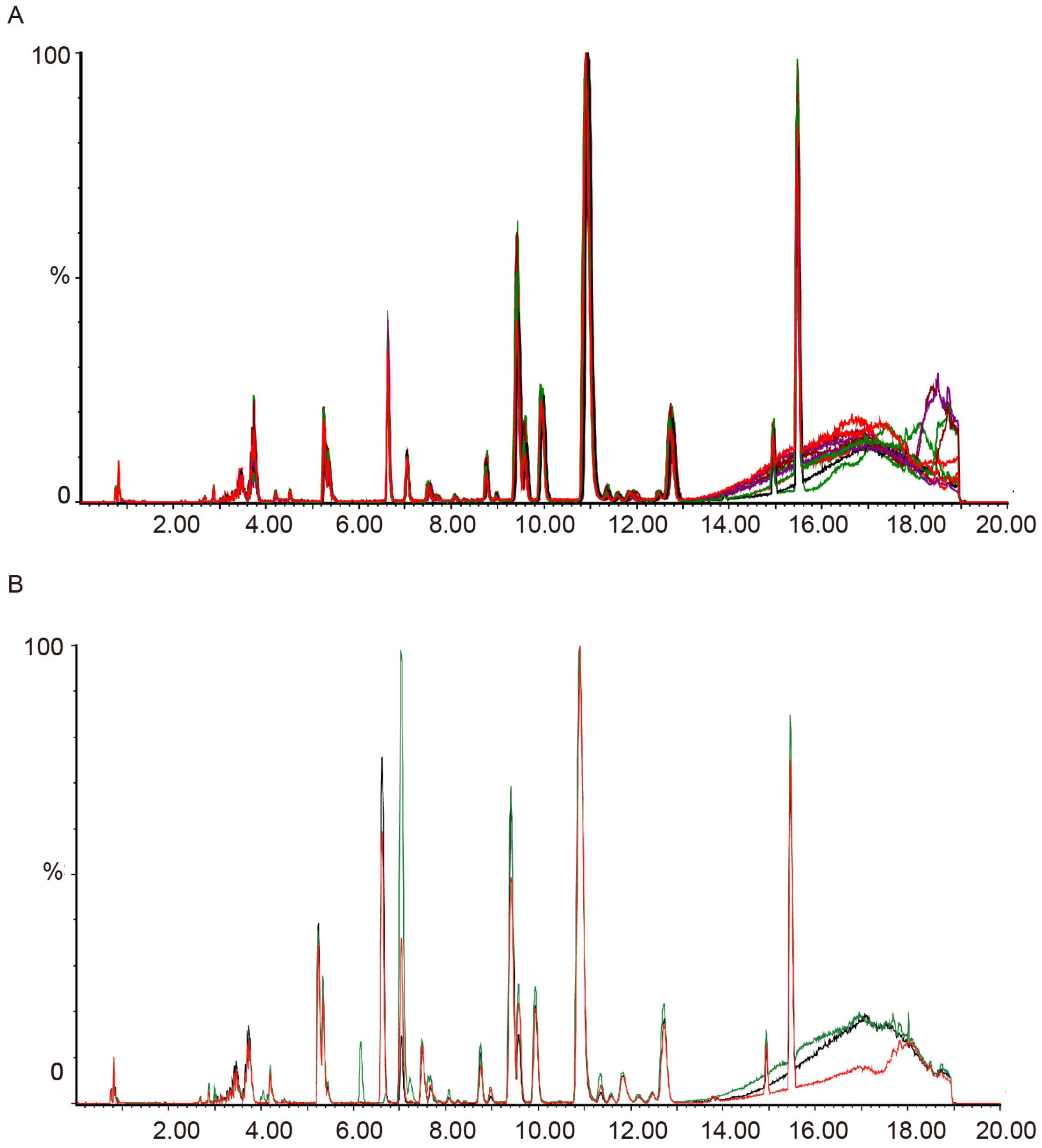

Fig. S1. A. Total ion current chromatograms of the QC samples. B. Typical UPLC-QTOF/MS base peak intensity (BPI) chromatograms for 1 NGR, 1 pre-DM and 1 DM sample in the positive mode. The Y axis shows relative abundance (%), and the X axis shows retention time in minutes, which was used to identify individual metabolites.

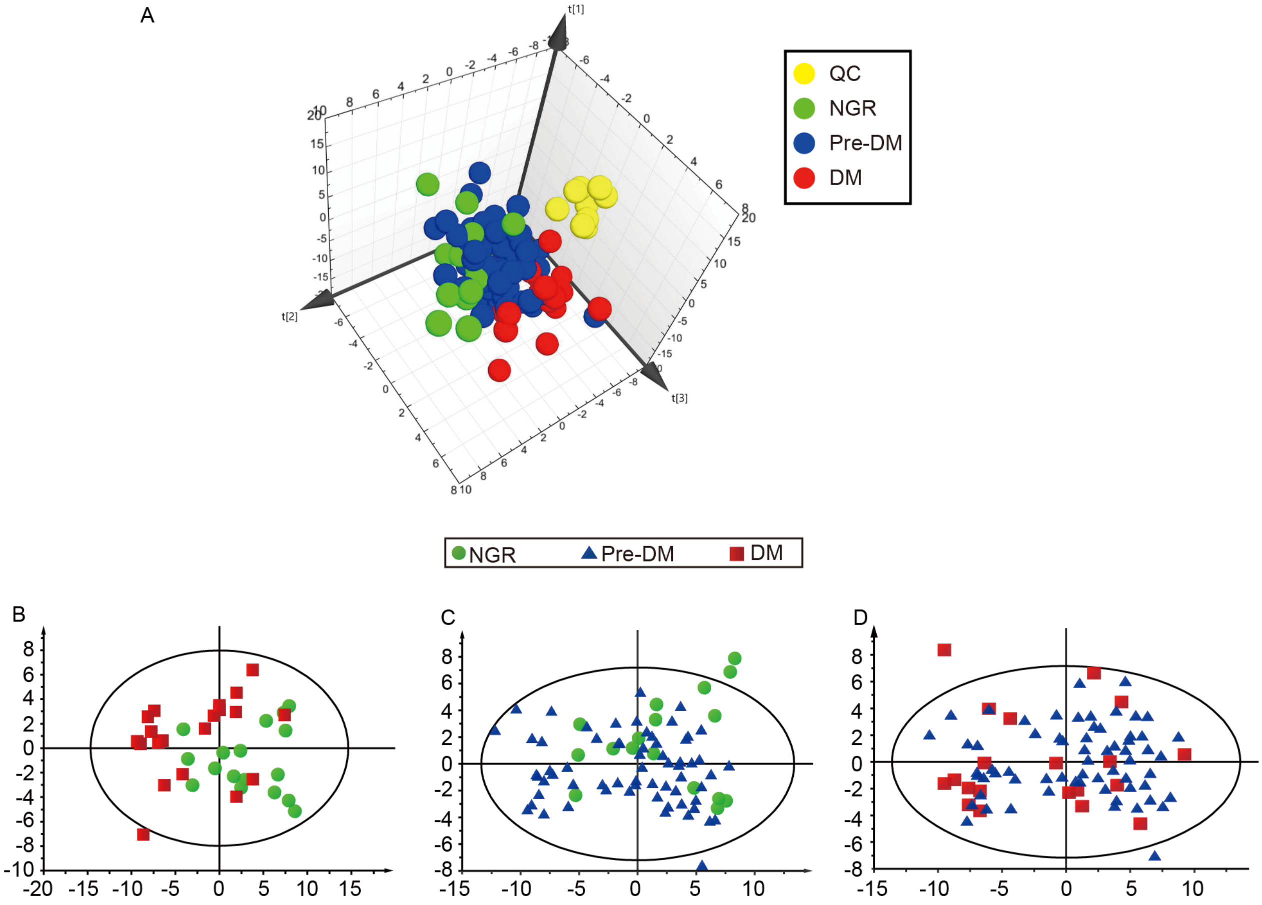

Fig. S2. PCA score plots of different groups based on plasma spectral data of UPLC-QTOF-MS positive ion mode. One point stands for one subject. A. PCA score plot of the NGR vs pre-DM vs DM groups and QC samples. B. PCA score plot of the the NGR vs DM. C. PCA score plot of the the NGR vs pre-DM. D. PCA score plot of the the pre-DM vs DM.

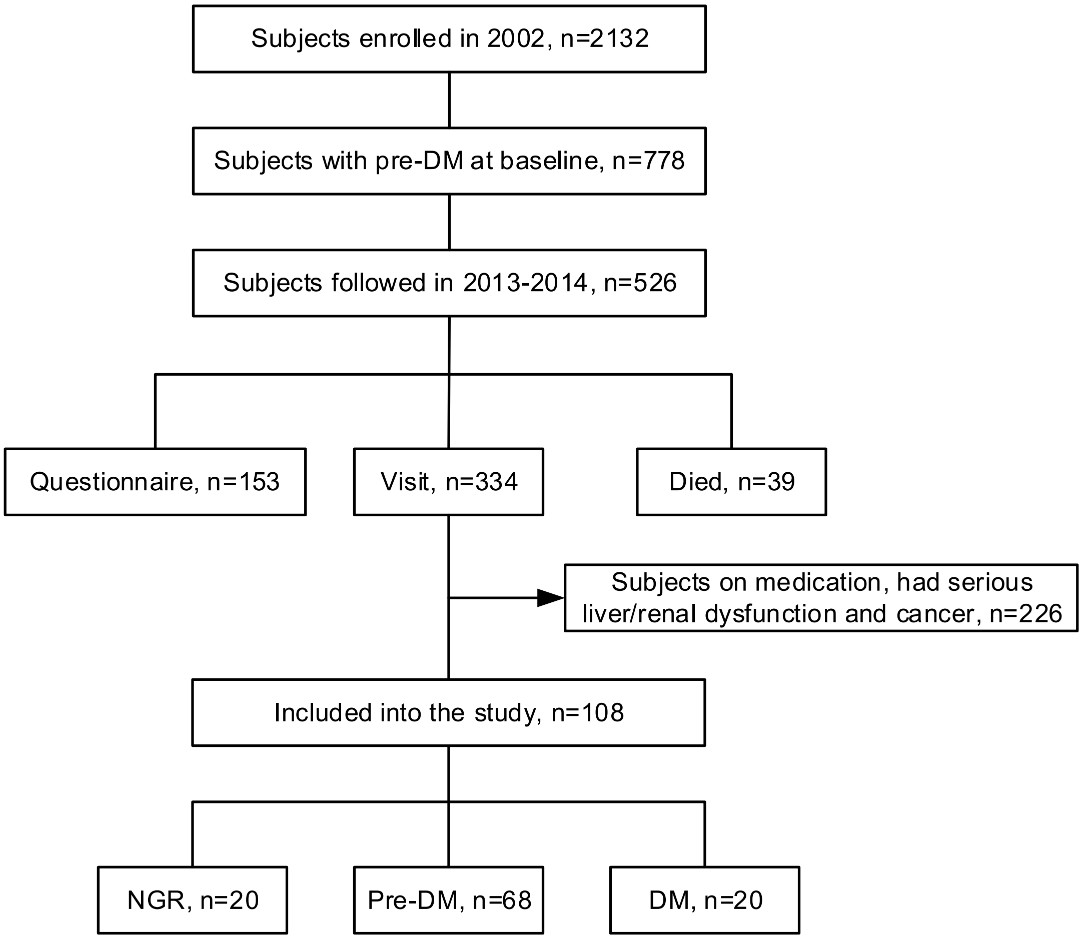

Fig. S3. Details on the study population.

	NGR(n=20)	Pre-DM(n=68)	DM(n=20)
Body mass index, kg/m^2	25.7±2.8	25.6±2.7	26.1±3.5
Waist circumference, cm	80.9±8.5	82.3±7.1	84.0±10.8
Waist-hip ratio	0.84±0.05	0.84±0.05	0.86±0.06
Hypertension, %	55.0	54.4	70.0
SBP, mmHg	134.8±16.9	134.5±16.6	135.3±22.7
DBP, mmHg	84.3±9.5	84.0±12.1	83.0±11.7
Fasting glucose, mmol/L	5.9±0.3	5.9±0.4	6.1±0.3
2-h glucose, mmol/L	5.7±0.8	5.9±1.3	6.0±1.5
HDL, mmol/L	1.4±0.3	1.4±0.3	1.5±0.4
LDL, mmol/L	3.0±0.8	2.9±0.7	3.1±0.9
TC, mmol/L	5.1±0.9	5.1±0.9	5.1±1.2
TG, mmol/L	1.4±0.8	1.7±1.1	1.8±1.1

Table S1. Characteristics of the study participants at baseline. Values are mean ± SD or %. Abbreviations are the same as Table 1.

Metabolites	AUC	Significance	95% CI		CV% in QC samples
			Lower	Upper	
20-Hydroxy-leukotriene E4	0.749	0.001	0.637	0.862	13.9
Delta 8,14 -Sterol	0.640	0.063	0.489	0.791	14.8
Pantetheine	0.743	0.001	0.606	0.879	18.5
Caprylic acid	0.670	0.024	0.538	0.802	10.6
N(6)-(octanoyl)lysine	0.665	0.028	0.515	0.816	11.3
Lysyl-Tyrosine	0.642	0.060	0.490	0.793	8.7
Pc(18:3/20:3)	0.639	0.065	0.487	0.792	14.8
LysoPE(20:5/0:0)	0.651	0.045	0.494	0.808	13.7
Lysopc(20:4)	0.429	0.349	0.262	0.597	14.2
2,3-Epoxymenaquinone	0.764	<0.001	0.654	0.873	11.1
Lysopc(18:3)	0.332	0.026	0.204	0.460	10.9
cis-13,16-Docosadienoic acid	0.724	0.003	0.582	0.866	11.0
S-(hydroxymethyl)glutathione	0.716	0.004	0.579	0.854	12.8
3-Phenylbutyric acid	0.661	0.033	0.509	0.813	12.6
1-Stearoylglycerophosphoglycerol	0.725	0.003	0.595	0.855	12.4
5-methoxytryptamine	0.717	0.004	0.575	0.858	10.6
Lysopc(20:5)	0.366	0.075	0.241	0.490	13.1
Endomorphin-1	0.727	0.003	0.596	0.859	11.8
L-palmitoylcarnitine	0.715	0.004	0.584	0.837	7.9
N6-Acetyl-L-lysine	0.644	0.056	0.491	0.798	4.1
Pc(14:1/16:1)	0.683	0.015	0.564	0.803	9.4
LysoPC(20:3)	0.381	0.115	0.253	0.509	9.1
3-Ethylphenol	0.699	0.008	0.571	0.827	6.8

Table S2. AUC of single biomarkers identified for transition to NGR, significance of ROC analysis, 95% CI and CV% values in QC samples.

Metabolites	AUC	Significance	95% CI		CV% in QC samples
			Lower	Upper	
2-ketobutyric acid	0.234	<0.001	0.107	0.362	14.6
Iso-valeraldehyde	0.183	<0.001	0.076	0.289	9.9
Betaine	0.246	0.001	0.118	0.373	13.3
Pantetheine	0.343	0.033	0.199	0.486	14.2
Uric acid	0.331	0.022	0.214	0.447	15.2
L-carnitine	0.243	0.001	0.117	0.368	13.6
L-threonine	0.204	<0.001	0.081	0.328	14.8
Lysope(16:0/0:0)	0.348	<0.040	0.224	0.472	10.0
Linoleic acid	0.671	0.021	0.536	0.806	7.7
Lysopc(18:1)	0.325	0.018	0.193	0.457	10.3
3-dehydroxycarnitine	0.703	0.006	0.575	0.832	5.7
Palmitic amide	0.367	0.073	0.234	0.500	10.9
3,5-dihydroxybenzoic acid	0.256	0.001	0.125	0.388	12.7
L-lysine	0.276	0.002	0.167	0.385	12.8
2-Pyrroloylglycine	0.292	0.005	0.160	0.424	10.0
Kynuramine	0.784	<0.001	0.661	0.908	16.1
Dityrosine	0.687	0.012	0.551	0.822	13.2
5-hydroxy-2-oxo-4-ureido-2,5-dihydro-1h-imidazole-5-carboxylate	0.225	<0.001	0.107	0.343	11.8
Lysopc(18:0)	0.376	0.094	0.238	0.514	13.3
Pc(18:0/18:2)	0.432	0.356	0.298	0.566	18.1
1,3,7-trimethyluric acid	0.699	0.007	0.579	0.820	11.4
Pc(16:0/14:0)	0.347	0.039	0.211	0.483	18.6

Table S3. AUC of single biomarkers identified for transition to DM, significance of ROC analysis, 95% CI and CV% in QC samples.
